# Aging Regulated Through a Stability Model of Insulin/Insulin Growth Factor Receptor Function

**DOI:** 10.3389/fendo.2021.649880

**Published:** 2021-03-11

**Authors:** Marc Tatar

**Affiliations:** Department of Ecology and Evolutionary Biology, Brown University, Providence, RI, United States

**Keywords:** aging, *Drosophila*, insulin receptor, insulin, IGF, reproduction, longevity, insulin resistance

## Abstract

Mutations of the insulin-like receptor in *Drosophila* extend lifespan. New research suggests this receptor operates in two modes. The first extends lifespan while slowing reproduction and reducing growth. The second strongly extends lifespan without impairing growth or reproduction; it confers longevity assurance. The mutation that confers longevity assurance resides in the kinase insert domain, which contains a potential SH2 binding site for substrate proteins. We apply a recent model for the function of receptor tyrosine kinases to propose how insulin receptor structure can modulate aging. This concept hypothesizes that strong insulin-like ligands promote phosphorylation of high threshold substrate binding sites to robustly induce reproduction, which impairs survival as a consequence of trade-offs. Lower levels of receptor stimulation provide less kinase dimer stability, which reduces reproduction and extends lifespan by avoiding reproductive costs. Environmental conditions that favor diapause alter the expression of insulin ligands to further repress the stability of the interacting kinase domains, block phosphorylation of low threshold substrates and thus induce a unique molecular program that confers longevity assurance. Mutations of the insulin receptor that block low-phosphorylation site interactions, such as within the kinase insert domain, can extend lifespan while maintaining overall dimer stability. These flies are long-lived while maintaining reproduction and growth. The kinase insert domain of *Drosophila* provides a novel avenue from which to seek signaling of the insulin/insulin-like growth factor system of humans that modulate aging without impacting reproduction and growth, or incurring insulin resistance pathology.

## Introduction

Mutations of the insulin/IGF tyrosine kinase receptor slow aging in *Drosophila* and *C. elegans*, and perhaps as well in humans (1–3). These invertebrates have single insulin/IGF-like receptors, InR in *Drosophila* and DAF-2 in *C. elegans*. Besides aging, these receptors regulate traits including development, growth, metabolism, reproduction, sleep, behavior, and Dauer/diapause (4–10). In mammals, a family of insulin, IGF, relaxin, and insulin-like peptides modulate many functions including metabolism, cell cycle, development, reproduction, cognition, and vascular physiology (11–13), where adult insulin and IGF1 signals *via* three dimeric receptors [IR, IGF1-R, IR/IGF1R hybrid (14)]. In contrast, the single invertebrate insulin-like receptors respond to a number of unique insulin-like ligands, seven in *Drosophila* and as many as 40 in *C. elegans* (15, 16). Despite their centrality, little is understood about how these invertebrate insulin-like ligands control such an array of distinct phenotypes. Here we explore a potential solution. We integrate new observations derived from single amino acid substitutions of *Drosophila* InR (17) with the receptor tyrosine kinase (RTK) threshold model of Zinkle and Mohammadi (18). We will propose that the level of insulin-stimulated dimer stability determines which substrate binding sites are activated to impact specific traits. Mutations of *InR* may slow aging because they reduce overall receptor dimer stability or because they directly modify binding sites. This model suggests how insulin-like receptors might slow aging without insulin-resistance and how diverse *Drosophila* insulin-like ligands control unique sets of traits. The model provides a framework to understand where and how modified insulin/IGF signaling can affect human aging.

## The Threshold Model of Receptor Tyrosine Kinase Signaling

Receptor tyrosine kinases (RTK) are single-pass transmembrane proteins that transduce extracellular ligand binding into kinase activity. Strongly bound ligands are thought to induce sustained kinase activity to promote outputs distinct from those of weak ligands, which produce transient or low kinase activity; the *intensity and duration* of intracellular signaling pathways determines the cellular response (19). As reviewed in Zinkle and Mohammadi (18), this process was first proposed for rat PC12 cells where the duration of MAPK activation differentially promotes neurite outgrowth *versus* cell proliferation, independent of ligand or receptor identity (19). In a second example, isoforms of fetal growth factor (FGF) ligand FGF8a and FGF8b differentially induce the midbrain to differentiate or expand. This specificity, however, is based on the relative abundance of each isoform and the associated magnitude of Ras/MAPK induction, not upon the ligand identity (20).

RTK also phosphorylate binding sites within their juxtamembrane (JM), C-terminal tail, and kinase domains. These sites recruit adapter proteins including those with Src homology 2 (SH2), phosphotyrosine-binding (PTB), and SH3 domain-binding sites. The identity of recruited substrate specifies which transduction pathways the receptor activates (21–23). Thus, mutation of one docking site can alter one particular outcome without affecting others, for instance when mutation of the Grb2-recruitment site on the canine kidney cell MET receptor blocks tubulogenesis without disrupting cell dissociation (24). In this view, the *quality* of the receptor-protein interaction determines the cellular response.

**Figure 1:**
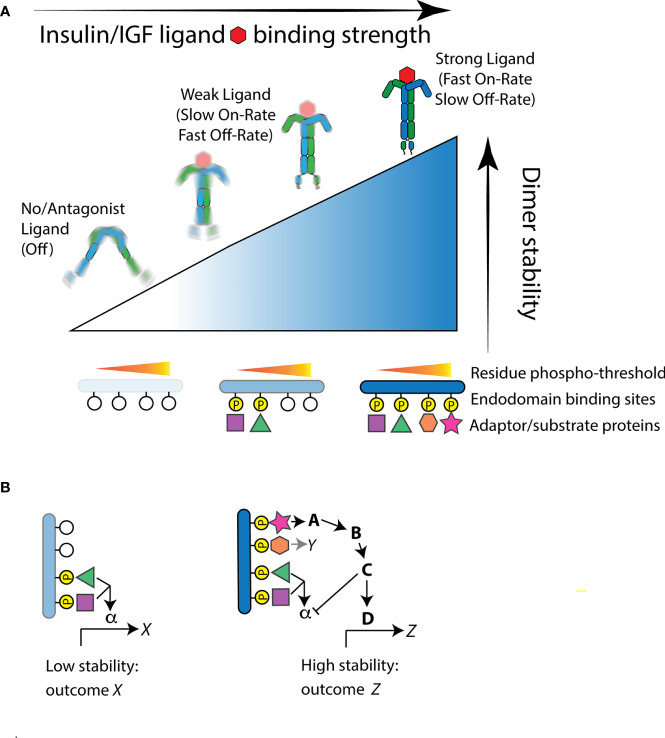
The Receptor Tyrosine Kinase threshold model of Zinkle and Mohammadi applied to the insulin-like receptor. **(A)** Structural reorientation of insulin-like protomers is induced by ligand binding. Strong binding ligands have fast-on/slow-off rates and produce highly stable interactions of the internal kinase domains. This permits phosphorylation of endodomain substrate binding sites that have high as well as low thresholds, thus recruiting a full complement of available substrate proteins. Weaker ligands produce moderate kinase domain interaction stability and thus only induce residue phosphorylation at sites with a relatively low stability threshold. In the absence of ligand, or when the receptor is bound by an antagonist ligand, protomers fail to reorient or are highly unstable. In this state, the kinase domains do not phosphorylate substrate residue sites and few if any substrate binding protein are engaged. **(B)** Signal feedback among hierarchical thresholds can produce unnested signaling outcomes. As an example: In weak stability activation of the receptor, low threshold binding protein interactions activate a signal pathway through the substrate protein α to induce a transcriptional program *X*. This program is not necessarily activated, however, when the receptor gains greater stability, even though the substrate protein α is recruited. A high threshold substrate interaction that activates binding protein A may simultaneously propagate signaling to induce the transcriptional program *Z* and repress signaling otherwise propagated by α.

Zinkle and Mohammadi (18) integrate how the *intensity of activation* and the *quality of interactions* determine RTK function. Ligand binding causes receptor tyrosine kinase protomers to dimerize or in the case of IR preformed dimers cause the intracellular domains to structurally reorient (25). Repositioning of IR intracellular domains is induced when insulin binds multiple ectodomain sites upon both protomers to affect hinge motions that bring each internal kinase domains into proximity, permitting them to asymmetrically transphosphorylate A-loop tyrosine residues (26–28). This transactivation stimulates subsequent kinase activity to phosphorylate endodomain tyrosine residues and substrate binding proteins. Central to the model (18), the level of stability between the repositioned intracellular domains determines which endodomain tyrosine residues are phosphorylated, where adaptor binding sites have unique phosphorylation thresholds. High affinity insulin ligands will have fast on-rates and slow off-rates at receptor binding sites and thus continuously stabilize the dimer to phosphorylate both low- and high-threshold sites (**Figure 1A**). Relatively weak or transient ligands will have slower on-rates and faster off-rates and consequently induce weak dimer stability that only activates binding sites with low phosphorylation thresholds. As a general point for the model relevant for any RTK, although thresholds are ordered, cellular responses need not be nested because signals from a high threshold site can inhibit the output from lower threshold sites (**Figure 1B**).

Overall, Zinkle and Mohammadi synthesize both perspectives of RTK operation: the intensity and duration of dimer stability regulates which binding proteins are activated, and these substrates specify the cellular outcome of the stimulated receptor. Here we develop how this threshold model helps explain control of aging by insulin-like receptors. First we describe longevity-extending mutations of *Drosophila InR* and *C. elegans daf-2*, and introduce known adaptor proteins of InR.

## The *Drosophila* and *C. elegans* Insulin-Like Receptors

Gems, Patel, and colleagues classified multiple mutations of the *C. elegans* insulin-like receptor *daf-2* (29, 30). “Class 1” mutants include substitutions in the extracellular CR, L2, and FnIII domains. These induce dauer, an alternative quiescent developmental stage, and promote adult longevity. “Class 2” substitutions reside in the L1 ligand pocket, the CR ectodomain, and the intracellular tyrosine kinase domain. These alleles induce dauer and extend lifespan, but also variously affect feeding, reproduction, movement, and growth (29). Class 1 and Class 2 alleles stimulate unique transcriptional profiles (30). To explain these differences, Patel (30) suggested Class 1 mutants reduce DAF-2 abundance and thus activate the transcription factor DAF-16/FOXO. Class 2 alleles were thought to increase receptor perdurance and thereby reduce interaction with Ras-associated substrates while retaining signal induction of PI3K/Akt. From extensive phenotypic analyses, these authors suggest the DAF-2 receptor has two distinct functional outputs.

**Figure 2:**
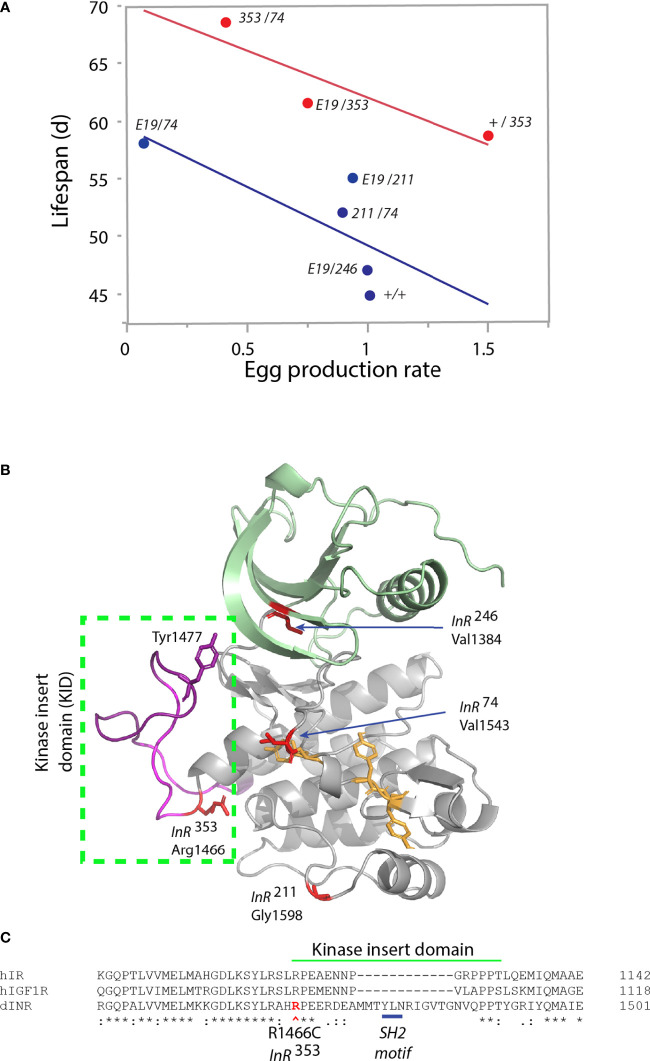
Characteristics of single amino acid substitution of the *Drosophila* insulin-like receptor. **(A)** Relationship of genotype average median lifespan relative to rate of egg production, from Yamamoto (17). Egg production rate is number of eggs produced daily scaled by the ovary size (number of ovarioles: ovary subunits). Lifespan is the average of median survival among replicate trials and independent genetic accessions of each genotype. Blue regression: genotypes lacking the *InR*^353^ allele (“353” in figure). Red regression: genotypes that include one *InR*^353^ allele. “E19” is the allele *InR*^E19^, a V*810*D substitution in the extracellular FnIII domain (17). Other alleles (*InR*^74^, *InR*^211^, *InR*^246^; in figure “74,” “211,” “246”) are substitutions within the kinase domain. The +/+ genotype is wildtype coisogenic will all mutant alleles, co-derived by homologous recombination; details in Yamamoto (17). **(B)** Model of kinase domain structure for the *Drosophila* insulin-like receptor. Subdomains: N-lobe (green), C-lobe (gray), A-loop tyrosines (yellow), Kinase Insert Domain (magenta) within box. Substitution residues for kinase domain mutations in red; Tyrosine*1477* of the proposed SH2 binding site within the KID. **(C)** Partial amino acid alignment of the C-lobe to include region of the KID in human IR, human IGF1R and *Drosophila* InR [alignments and nomenclature from (17)]. Site of the *InR*^353^ substitution in red (R*1466*C), site of the proposed SH2 motif underlined in blue.

We recently studied how mutations in *Drosophila InR* affect aging (17). InR is generated from three alternative 5’UTRs (31, 32) to produce isoforms differing by a 368-amino acid C-terminal tail (33–35). Based on our analysis of codon substitutions, InR appears to modulate aging through distinct modes (**Table 1**). As transheterozygotes, Mode 1 alleles increase survival, decrease egg production, reduce body size, and repress insulin-stimulated Akt phosphorylation (17). Among genotypes from these alleles, lifespan negatively correlates with egg production (**Figure 2A**), consistent with theory for how aging arises when selection optimizes fitness (47). These pro-longevity mutations produce amino acid substitutions in the extracellular FnIII domain (extracellular V*810*D), and in conserved residues of the kinase A-loop and the kinase C-lobe (**Figure 2B**). As a group, these substitutions are likely to destabilize protomer endodomain interaction or directly inhibit kinase catalytic function (27, 28).

**Table 1:** Phenotypes of *Drosophila* insulin/IGF receptor and substrate protein mutations.

Genotype	Lifespan increaseDays (proportion)	Net fecundity, proportion	Adult size, proportion	Ref
***Mode 2: Increase longevity without reduced fecundity or growth***				
**WT/*InR*^353^**	10–16 d (1.2–1.4)	1.6	1.0	(17)
**WT/*chico*^1^**	14–18 d (1.3–1.4) 10 d (1.4) 3–16 d (1.1–1.4) 12–22 d (1.2–1.5) 8 d (1.1) 10 d (1.2)	2.0 0.80	1.0 1.0	(36) (37) (38) (39) (40) (41)
***Mode 1: Increase longevity with reduced fecundity or growth***				
***InR*^74^, *InR*^E19^, *InR*^211^**	6–14 d (1.2–1.4)	0.05–0.75	0.81–0.88	(17)
***chico*^1^/*chico*^1^**	16 d (1.3) 16 d (1.6) 12–22 d (1.2–1.5) 18 d (1.4)	sterile	0.40–0.50 0.35	(36) (37) (38) (42) (41)
**Lnk/Lnk** **(SH2B1)**	5–8d (1.0–1.1)	<0.2	0.60–0.65	(43) (44) (45)
**InR-DN**	9–13 d (1.2–1.4)	0.19–0.86	0.55	(46)
**UAS-p110**	5 d (1.1)	0.73		(46)

Compiled from sources that together describe lifespan and reproduction (female); and adult size when available. Values for lifespan are the average gain in median survival relative to wildtype controls, in days and as a proportion relative to control. When shown, range is among replicate trials within the publication. Fecundity: net egg production per female across the measured duration of each genotype relative to wildtype. Adult size based on mass or wing area, as a proportion relative to wildtype. Empty cells: data not available. Upper table compiles **Mode 2** genotypes: longevity is extended without reduced fecundity or impaired growth; representing longevity assurance. Lower table compiles **Mode 1** genotypes: longevity is extended while reproduction and growth are impaired; representing life history trade-offs.

Mode 2 is represented by the dominant allele *InR*^353^ (17). Adult heterozygotes (wildtype/*InR*^353^) have robustly increased lifespan but remarkably so without decreasing reproduction or growth (**Table 1**). Unlike Mode 1 flies, tissue from the *InR*^353^ heterozygotes strongly induces pAkt in response to insulin—they are not insulin resistant. When *InR*^353^ is combined with Mode 1 alleles, adults lay fewer eggs and lifespan is increased by the combined effects of reduced survival costs of reproduction added to the longevity assured by *InR*^353^ (**Figure 2A**).

The *InR*^353^ substitution Arg*1466*Cys lies within the kinase insert domain (KID) (**Figure 2C**), an unstructured peptide segment that interrupts the kinase domain of many RTKs (48). Arg*1466* of Drosophila is homologous to Arg*1092* of the human insulin receptor (49). In humans, the insulin receptor mutation Arg*1092*Glu produces Donohue syndrome where heterozygotes are largely normal while homozygotes are strongly insulin resistant, small, and inviable (50). Overall, the function of kinase insert domains is poorly understood but whereas the human IR and IGFR domains are short, the longer *Drosophila* KID contains a potential SH2 binding motif (Tyr*1477*-Leu-Asn; **Figure 2C**). This site may recruit an adaptor protein, potentially Grb2 as seen in the KID of mammalian PDGFR, CSF1R, and Kit (48). We hypothesize the *InR*^353^ substitution disrupts this receptor-protein interaction to induce longevity assurance—a homeostatic program that increases somatic survival independent of reproductive trade-offs (51).

## Adaptor and Substrate Proteins of *Drosophila* InR

A number of receptor-adaptor protein interactions are documented for the *Drosophila* insulin receptor. The C-terminal tail of InR recruits Chico (homolog of IRS1-4), although apparently without phosphorylating this substrate (52, 53). The tail likewise contains Y*XX*M motifs to recruit the p85/p60 subunit of PI3-kinase (34), and P*XX*P sequences for the SH2/SH3 adapter Dock (homolog of mammalian Nck) (54). Dock modulates photoreceptor axon guidance but does not affect growth. No data address if protein interactions with the C-terminal tail affect aging.

The juxtamembrane domain (JM) of InR also recruits Chico, using NP*X*Y residues conserved in the human insulin receptor (42, 53). Interaction between InR and Chico is mediated by the SH2B1 adaptor protein Lnk (44, 45, 55). In mammals, SH2B1 is recruited to insulin receptor A-loop phosphotyrosines (56, 57). In *Drosophila*, Lnk colocalizes InR and Chico to promote phosphorylation of Akt (55). Genetic loss of Lnk extends longevity, reduces body size, and represses fecundity (**Table 1**).

Mutation of *chico* itself slows aging (**Table 1**). Appropriate for the centennial of insulin discovery, *chico* is debated to harken back to 1919, potentially as an allele of the mutation *flipper* identified by Bridges and Mohr (see https://flybase.org/reports/FBgn0000675). Modern *chico* mutant alleles are transposon insertions initially characterized to elevate lipids, and impair cell size and number (42, 58). Homozygotes of the mutant *chico*^1^ are small, long-lived, and sterile; wildtype/*chico*^1^ heterozygotes are also long-lived and similar to wildtype/*InR*^353^ these adults have normal growth and fertility (36–38) (**Table 1**).

Chico is a substrate adapter protein. It recruits SH2/SH3 domain-containing proteins including the p85/p60 subunit of PI3K and the Grb2 homolog Drk (Downstream of receptor kinase) (42, 53, 59). Oldham expressed *chico*-transgenes in *chico*^1^ homozygotes (59). Wildtype *chico*-transgenes rescued body size and fertility. Transgenes that only restored Grb2/Drk binding did not rescue these traits while those that restored p60/PI3K restored growth and reproduction. Slack (40) used this design to study aging. The exceptional longevity of *chico*^1^ heterozygotes reverted to normal by addition of a wildtype *chico* transgene but not when the *chico* transgene contained only functional p60/PI3K sites or only functional Grb2/Drk sites. Overall, Chico controls p60/PI3K/Akt to modulate growth, metabolism, and longevity, but its effects through Grb2/Drk appear to be limited to aging.

As in mammals, activated InR phosphorylates Akt to repress *Drosophila* Foxo, the homolog of mammalian FOXO1-4 and *C. elegans* DAF-16. As seen for *daf-16*, *foxo* is required for insulin receptor mutations to extend *Drosophila* lifespan (39, 46). Gene targets of these transcription factors in both invertebrates reveal many distal mechanisms to slow aging (60–62). Parallel to Akt-Foxo, Drosophila Grb2/Drk regulates Ras to control signaling through Erk (63). Slack (40) demonstrated Chico acts through Ras-Erk to regulate the E-twenty-six transcription factor Anterior Open (Aop). Aop is required for *chico* mutations to extend lifespan, however no data yet shows if this interaction is downstream of InR rather than other potential IRS-regulatory receptors (64).

These observations provide three touchpoints. First, mutations of *InR* may affect aging through altered kinase activity while another may act by altering adapter protein interaction. Second, *InR*^353^ and *chico*^1^ are dominant alleles that produce long-lived adults that are unexpectedly large and fecund. Third, Chico appears to signal through SH2-Grb2/Drk-Ras to modulate aging without affecting growth or reproduction, while we suggest the InR kinase insert domain contains an unrecognized SH2 binding motif. The Arg*1466*Cys substitution of *InR*^353^ within the KID may destabilize Grb2/Drk direct signaling to slow aging. These observations can be integrated with the RTK threshold model to hypothesize how InR regulates aging.

## Hypothesis: Stability Thresholds to Regulate Aging

**Figure 3:**
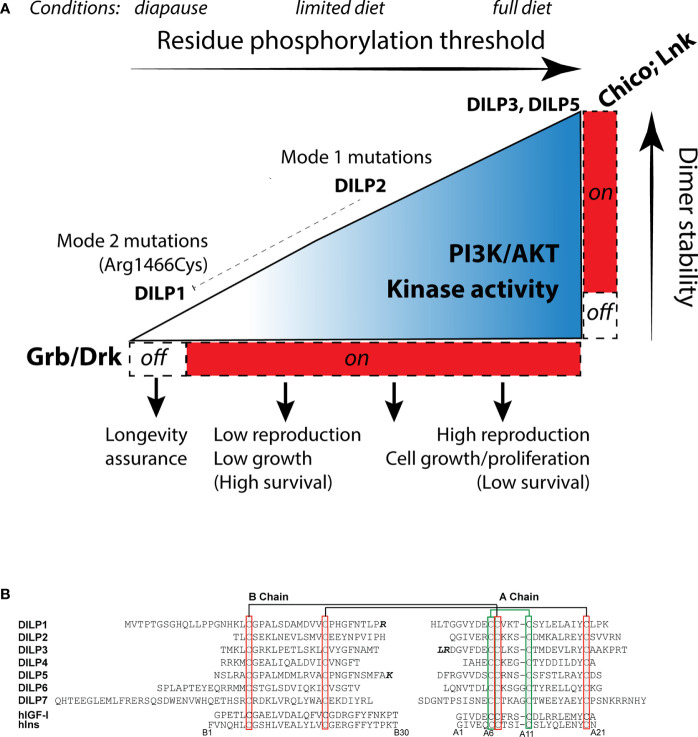
How insulin-like receptors and ligands may modulate aging relative to reproduction, growth and metabolism through the Receptor Tyrosine Kinase threshold model. **(A)** The environment determines the level of insulin-like receptor dimer stability through control of *Drosophila* insulin like peptides (DILP). Peptides with high binding activity (DILP5, perhaps DILP3) promote stable protomer kinase interaction, leading to strong kinase catalytic activity and phosphorylation of high threshold substrate binding sites, activation of the adaptor proteins LNK, Chico and Grb, and signal transduction through AKT and Ras. This stimulates reproduction and growth. Limited diet (or moderating environments) reduce DILP5 and DILP3 but retain DILP2 expression (65). DILP2 has reduced kinase dimer stability, potentially sufficient to activate AKT and RAS through PI3K and Grb/Drk recruitment but with less kinase catalytic activity. This state sustains less reproduction. DILP2 represses expression of *dilp1*. In extreme environments, flies enter diapause and express *dilp1*. We propose DILP1 is a competitive receptor antagonist. Kinase dimer stability is minimized. High/moderate threshold sites required to activate Akt are not phosphorylated, ceasing reproduction. Low threshold sites required to activate Grb/Drk are not activated. This impairs Ras signaling, which induces systems to support somatic survival (longevity assurance). This state produces reproductive diapause. The *InR*^353^ mutation (Arg*1466*Cys), we propose, inhibits phosphorylation of Grb/Drk by the KID but does not (as observed) affect the ability of the receptor to induce phosphorylation of Akt. The mutation unleashes the longevity assurance program of diapause while bypassing the loss of dimer stability that would otherwise inhibit reproduction. **(B)** Sequences of the *Drosophila* insulin-like peptides DILP1-7, B- and A-chains, using cleavage sites predicted by Gronke (66), with potential alternatives where the additional residues are noted in bold. Chains are aligned across bridge cysteines. Human insulin and insulin growth factor 1 for comparison, numbered from insulin.

Zinkle and Mohammadi (18) propose stimulated RTK have varied levels of dimer stability that progressively phosphorylate adaptor binding sites, each with a characteristic threshold. Activated binding sites interact with specific adaptor proteins to stimulate unique cellular outcomes. We envision this model operates within insulin-like receptors (**Figure 3A**). In *Drosophila* InR, sites with high thresholds may include those that recruit Lnk and Chico while sites with a relatively Low phosphorylation threshold might recruit Grb2/Drk. In conditions favoring full reproduction, abundant, strong insulin ligands interact with InR to stabilize protomer kinase domain interaction. Strong transphosphorylation and extensive kinase activity phosphorylate both low (Grb2/Drk) and high threshold receptor binding sites (Chico, Lnk), and efficiently phosphorylate substrate proteins. The activated substrates transduce signals through Akt, TOR, Ras, AMPK, and GSK to promote growth and reproduction. These conditions are permissive for aging because lifetime reproductive success is optimized through the balance of egg production with associated survival costs.

In restricted conditions such as limited diet, adults secrete fewer or different DILPs (65). We propose this moderately reduces dimer stability to a level that dampens kinase activity while Akt is still phosphorylated. The receptor propagates less intense signaling, which reduces reproduction and correspondingly increases survival. In extreme conditions, such a season that induces diapause, we propose the endocrine state minimizes InR dimer stability so that low threshold residues become dephosphorylated. The SH2 motif of the kinase insert domain may represent such a site. It may be activated in normal conditions by insulin ligands to induce Grb2/Drk-Ras/Erk signaling. However, in diapause conditions key insulin ligands are repressed (67). We hypothesize this will destabilize InR dimers to dephosphorylate the SH2/Grb/Drk site of the KID, and thereby blunt Erk signaling to release somatic maintenance programs that retards somatic aging. Because of the hierarchy within the threshold model, high-phosphorylation threshold sites of InR will not be activated in this state of low dimer stability; Akt will not transduce pro-reproductive signaling. This mechanism models InR regulation of reproductive diapause; it simultaneously stalls reproduction and assures somatic survival until favorable environmental conditions return (68, 69).

This model may explain how some insulin receptor mutations slow aging without affecting reproduction or insulin sensitivity (Mode 2). We hypothesize the Arg*1466*Cyr substitution disrupts how Grb/Drk is recruited to the SH2 binding motif of the KID. This mutation, however, does not destabilize the dimer and heterozygous receptors therefore phosphorylate Akt and retain kinase activity that propagate reproduction and growth. Although, balancing this hypothesis, the drug Trametinib, a selective MEK1 and MEK2 inhibitor, extends fly lifespan while reducing fecundity (70). In contrast, Mode 1 mutations have reduced kinase activity and are therefore insulin resistant (17). We propose these mutations somewhat increase dimer instability, but not to an extent that dephosphorylates Try*1477*. Fecundity and growth are reduced with moderate loss of dimer stability, and longevity is increased by mitigating survival costs of reproduction.

## *Drosophila* Insulin-Like Ligands

In this threshold model, receptor dimer stability will be modulated by the quantity, quality, and bioavailability of insulin-like ligands. *Drosophila* has seven insulin-like loci, *dilp1-7* (49, 66, 71). Based on *dilp* sequence from 12 *Drosophila* species, Gronke (66) concluded these ligand peptides contain conserved cysteine disulfide bridges, bioactive A and B chains, and functional signal peptides (**Figure 3B**). DILP1, DILP6, and DILP7 are notable for their extended B-chain N termini. DILP6 has a short C-peptide sequence and may thus more resemble mammalian IGF. An alternative insulin-like peptide was subsequently identified, *dilp8*, which encodes a relaxin-like ligand that stimulates G protein-coupled signaling (72, 73). The insulin-like peptide genes are expressed in varied tissues from embryo to adult, and early work showed mutants of these loci affect growth and metabolism (49, 74, 75). In normal adults, *dilp2*-*3*, *and 5* are primarily produced in median neurosecretory cells (MNC) where they are released into the brain, into secondary endocrine organs, and into circulation (74). In contrast, adult *dilp1* is only expressed in MNC during reproductive diapause (76). The MNCs derive from anterior neuroectoderm of the fly embryo, orthologous to vertebrate adenohypophyseal placoid that is the developmental source of mammalian islet-like endocrine cells (77). As well, *dilp6* is expressed in the fat body, a tissue with liver- and adipose-related function (78, 79).

Synthetic and recombinant peptides have been used to reveal the function of individual DILPs. Dimeric recombinant DILP5 binds human insulin receptors in a manner consistent with negative cooperativity (80), and when injected into rats and Drosophila the recombinant hormone transiently lowers circulating sugar. DILP5 also interacts with the insect-binding protein Imp-L2 (80, 81), likely to antagonize circulating insulin (82, 83). Notably, elevated Imp-L2 is associated with extended lifespan, even in conditions where *dilp2*, *dilp3*, and *dilp5* mRNA are elevated (84–86). It is not known which insulins aside from DILP5 bind to Imp-L2.

A synthetic DILP2 was compared to DILP5 when these peptides stimulated Drosophila S2 cells in culture (87). These peptides induced broadly similar signaling elements (Akt, Erk, S6K) and transcriptional profiles, but they also revealed unique outputs. DILP5 produced high, continuous phosphorylation of Akt whereas DILP2 only induced a transient response. In a phosphoproteomic scan, DILP2 equally increased and decreased the number of total phosphorylation sites while DILP5 overwhelmingly increased total phosphorylation. Several specific proteins were differentially phosphorylated by these peptides. Notably, glycogen phosphorylase did not respond to DILP5 but the enzyme was dephosphorylated and inactivated in cells stimulated by DILP2, a response typical for human insulin. Conversely, elevated glycogen phosphorylase activity was found in *dilp2* mutant flies, which are long lived, while transgenic expression of *GlyP* was sufficient to extend lifespan. These data demonstrate measurable differences among specific DILPs acting through a common receptor. And they remind us that the action of insulin-like peptides in aging can involve non-genomic, cellular metabolic regulation independent of canonical FOXO transcription factors.

Understanding DILP function *in vivo* is complicated because mutation of one *dilp* changes the expression of others (66). Nonetheless, abundant data shows longevity is extended when *dilp2* is reduced alone or with other insulins (66, 79, 88). *dilp2* expression in adults is greatest on diet of low protein and high sugar (65). In contrast, adult *dilp1* is absent under normal conditions soon after eclosion, but is elevated 14-fold in *dilp2* mutants and 4-fold during diapause (76, 89). Post (89) demonstrated that dilp1 is required for loss of dilp2 to extend lifespan, but dilp1 is not required for the loss of dilp2 to induce dilp3 and dilp5 or stimulate phosphorylation of Akt. In contrast, loss of *dilp2* represses pErk in a *dilp1* dependent manner. DILP1 and DILP2 appear to have countervailing functions associated with diapause, longevity, and Erk signaling.

These observations suggest how Drosophila insulin-like peptides might regulate the outcomes of InR. We tentatively propose DILP5 (and perhaps DILP3) strongly stabilizes InR dimers; DILP2 transiently stabilizes the dimer; DILP1 inhibits InR stability and competitively blocks other insulin-like ligands. In good environments, DILP2, DILP3, and DILP5 promote dimer stability and kinase activity. This activates pAKT and pERK signal transduction to promote growth and reproduction. In this state DILP2 simultaneously represses *dilp1*. Conditions of limited diet repress *dilp3* and *dilp5* but not *dilp2* (65); dimer stability is moderately reduced. This state still phosphorylates Akt but diminishes kinase signaling, which down-regulates reproduction and improves survival. At the extreme, in diapause, *dilp1* is transcribed. Abundant DILP1 inhibits the binding of other insulin ligands to the receptor, minimizes dimer stability, prevents Akt phosphorylation to retard reproduction, and extinguishes Grb2/Drk-Erk signaling to induce systems of longevity assurance.

This sketch is speculative and incomplete. No work yet reveals how DILP1 or DILP2 interact with InR, or how any DILP affects dimer stability or substrate protein interaction. We have not considered DILP6, perhaps the most IGF-like fly ligand, which non-autonomous affects aging through its action in the fat body (78, 79). Little functional data are available for DILP3 despite its abundance in adults. There is much work ahead.

## The Paradox Of Insulin Resistance and Longevity

How could altered insulin-like signaling support healthy human aging as found in *C. elegans* and *Drosophila*? One solution argues the domain-defined functions of the invertebrate insulin-like receptor are distributed across the mammalian IR and IGFR receptors. The Arg*1466*Cys substitution of the *Drosophila* kinase insert domain promotes longevity without impairing growth and reproduction, or incurring loss of kinase activity (stimulated pAkt). Similar outcomes arise in *chico* heterozygotes and when the SH2/Grb site of Chico is blocked. None of these genotypes are particularly hyperglycemic or insulin resistant (17, 40). Instead, insulin resistance occurs in InR genotypes where we predict the mutations reduce stability of activated protomers. These outcomes suggest we identify where the longevity assurance function of the *Drosophila* KID translates to human IR or IGFR. While the human kinase insert domains share the KID sequence Arg-Pro-Glu where Arg*1466*Cys is substituted in Drosophila *InR*^353^, the human KID are small and lack the SH2 motif proposed for *Drosophila*. It is possible in the evolution of insulin-like receptors that some ancestral KID functions were integrated into the four insulin receptor substrates of mammals, as may also be the case of the *Drosophila* IRS-like C-terminal tail. In particular, IRS2 contains SH2 binding motifs that recruit Grb2, and mice mutant for IRS2 are long-lived (90). This property of IRS2 could involve interactions with IGF1R (91, 92). Notably, human polymorphisms in *IGF1R* are associated with survival to extreme age (3), and mice heterozygous for *IGF1R* are long-lived in some genetic backgrounds (93). It would be interesting to explore how these *IGF1R* genotypes affect specific phospho-sites of IRS2, and whether they alter Grb/Ras/Erk signaling.

If human aging can be modulated by IGFR-IRS2, insulin resistance is not required to slow aging (94), which is otherwise a paradox attributable to invertebrate models. Insulin resistance and slow aging indeed covary in *Drosophila* but the traits are decoupled in a mutation of the KID that potentially avoids loss of high dimer-stability signaling. Parallel benefits in humans might occur through elements of IGFR-mediated signaling rather than through reduced insulin sensitivity.

## Data Availability Statement

The raw data supporting the conclusions of this article will be made available by the author, without undue reservation.

## Author Contributions

The author confirms being the sole contributor of this work and has approved it for publication.

## Funding

MT has been supported to study the role of insulin signaling in aging by the National Institutes of Health through awards NIH R01AG16632 and R37AG024360.

## Conflict of Interest

The author declares that the research was conducted in the absence of any commercial or financial relationships that could be construed as a potential conflict of interest.
